# Tunable Synthesis of Mesoporous Carbons from Fe_3_O(BDC)_3_ for Chloramphenicol Antibiotic Remediation

**DOI:** 10.3390/nano9020237

**Published:** 2019-02-10

**Authors:** Thuan Van Tran, Duyen Thi Cam Nguyen, Hanh T. N. Le, Long Giang Bach, Dai-Viet N. Vo, Seong Soo Hong, Tri-Quang T. Phan, Trinh Duy Nguyen

**Affiliations:** 1 Center of Excellence for Green Energy and Environmental Nanomaterials (CE@GrEEN), Nguyen Tat Thanh University, 300A Nguyen Tat Thanh, District 4, Ho Chi Minh City 755414, Vietnam; tranuv@gmail.com (T.V.T.); ntcamduyen@gmail.com (D.T.C.N.); vietvo@ump.edu.my (D.-V.N.V.); pttquang@ntt.edu.vn (T.-Q.T.P.); 2 NTT Hi-Tech Institute, Nguyen Tat Thanh University, 300A Nguyen Tat Thanh, District 4, Ho Chi Minh City 755414, Vietnam; blgiang@ntt.edu.vn; 3 Department of Pharmacy, Nguyen Tat Thanh University, 298–300A Nguyen Tat Thanh, Ward 13, District 4, Ho Chi Minh City 700000, Vietnam; 4 Institute of Hygiene and Public Health, 159 Hung Phu, Ward 8, District 8, Ho Chi Minh City 700000, Vietnam; lethingochanh@iph.org.vn; 5 Center of Excellence for Functional Polymers and NanoEngineering, Nguyen Tat Thanh University, 300A Nguyen Tat Thanh, District 4, Ho Chi Minh City 755414, Vietnam; 6 Faculty of Chemical & Natural Resources Engineering, Universiti Malaysia Pahang, Lebuhraya Tun Razak, 26300 Gambang, Kuantan, Pahang, Malaysia; 7 Department of Chemical Engineering, Pukyong National University, 365 Shinsunro, Nam-ku, 48547 Busan, Korea; sshong@pknu.ac.kr

**Keywords:** removal of chloramphenicol antibiotic, metal–organic frameworks, porous carbon

## Abstract

Chloramphenicol (CAP) is commonly employed in veterinary clinics, but illegal and uncontrollable consumption can result in its potential contamination in environmental soil, and aquatic matrix, and thereby, regenerating microbial resistance, and antibiotic-resistant genes. Adsorption by efficient, and recyclable adsorbents such as mesoporous carbons (MPCs) is commonly regarded as a “green and sustainable” approach. Herein, the MPCs were facilely synthesized via the pyrolysis of the metal–organic framework Fe_3_O(BDC)_3_ with calcination temperatures (*x* °C) between 600 and 900 °C under nitrogen atmosphere. The characterization results pointed out mesoporous carbon matrix (MPC700) coating zero-valent iron particles with high surface area (~225 m^2^/g). Also, significant investigations including fabrication condition, CAP concentration, effect of pH, dosage, and ionic strength on the absorptive removal of CAP were systematically studied. The optimal conditions consisted of pH = 6, concentration 10 mg/L and dose 0.5 g/L for the highest chloramphenicol removal efficiency at nearly 100% after 4 h. Furthermore, the nonlinear kinetic and isotherm adsorption studies revealed the monolayer adsorption behavior of CAP onto MPC700 and Fe_3_O(BDC)_3_ materials via chemisorption, while the thermodynamic studies implied that the adsorption of CAP was a spontaneous process. Finally, adsorption mechanism including H-bonding, electrostatic attraction, π–π interaction, and metal–bridging interaction was proposed to elucidate how chloramphenicol molecules were adsorbed on the surface of materials. With excellent maximum adsorption capacity (96.3 mg/g), high stability, and good recyclability (4 cycles), the MPC700 nanocomposite could be utilized as a promising alternative for decontamination of chloramphenicol antibiotic from wastewater.

## 1. Introduction 

Antibiotics have a great contribution to defending the human immune system from pathogens involved in microorganisms, thereby, they have been artificially synthesized to satisfy the demands for treatment [1,2,3]. Among them, chloramphenicol (CAP) is commonly employed in veterinary clinics. Due to its severe genotoxicity and side-effects such as leukopenia and agranulocytosis, CAP utilization is restrained, even banned nowadays [4]. Unfortunately, its cost-effectiveness and widespread availability have driven CAP pharmaceutical to be produced illegally and used uncontrollably, causing their potential contamination towards environmental soil, conspicuously in the aquatic matrix, also, regenerating microbial resistance, and antibiotic-resistant genes [5].

**Figure 1:**
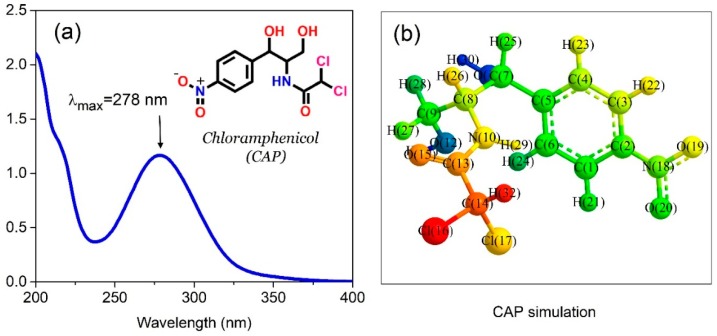
UV-Vis spectra (**a**) and structural formula (**b**) of chloramphenicol (CAP) simulated by molecular dynamics from the Chem3D program.

Generally, CAP molecules are chemically chlorinated via a complex string of dihydroxylation, reduction and addition steps initiated by nitroaromatic derivatives (Figure 1). Table 1 summarizes its properties, which consist of functional groups such as nitro, hydroxyl, and amide, probably forming total 9 H-bonds (3 H-acceptor and 6 H-donors). CAP substance in aqueous solvents presents a maximum absorption wavelength at 278 nm (Figure 1). Otherwise, this compound exhibits a strong solubility in water (2.5 g/L) according to Merck Index (2001) and offers a high degree of hydrophilicity with relatively low log Kow (1.14), and molecular polarity as illustrated in Figure 1b. During the gradually enzymatic biodegradation in the metabolic system, substituted chlorines have a wide range of detrimental effects on habitats [6]. Several studies have indicated that the biological remediation of such pollutants is benign, but inefficient [7]. There appears to be a tendency for technologies to acquire the primary requirements for antibiotic treatment in terms of performance, removal efficiency and cost [8,9,10]. Satisfying these criteria, adsorption is accessible as a “green and sustainable” approach derived from recyclable and effective adsorbents including mesoporous carbons (MPCs) [11,12,13].

The metal–organic frameworks (MOFs) are structurally constructed from metal clusters and organic linkers, they are impressive for their diverse application in many fields as catalysis, adsorption, drug delivery and gas adsorption [14,15,16,17]. The topology of Fe_3_O(BDC)_3_ (Fe-MIL-88B) with iron metal sites connected with 1,4–dicarboxylic acid (H_2_BDC), is representative for the synthesis of MPCs since it possesses an abundance of precious characteristics, particularly in chemical stability, as well as facile synthesis via hydrothermal methods [18,19,20]. Structurally, the features of Fe_3_O(BDC)_3_ rely on exceptional adaptability and flexibility of its nanoscale pore against guest species [21]. Therefore, the Fe_3_O(BDC)_3_ structure has brought a bunch of significant applications, such as Fenton photocatalytic activity in organic dye degradation and arsenic remediation [21].

Tunable transformation of Fe-based MOFs into magnetically and hierarchically mesoporous MPCs has recently reported [22,23,24,25]. Direct conversion of these MOFs can be followed by heat treatment, which pore sizes, morphologies, compositions, and properties of MPCs are not only partially inherited from MOF precursors, but also dependent on temperature control [26]. Ming Hu et al. reported a simple pyrolysis for the fabrication of nanoporous carbons with a very high surface area (5500 m^2^/g) along with large pore sizes, hierarchical morphologies, diverse functional groups exhibiting superior sensing capabilities toward toxic aromatic substances [27]. The pyrolysis strategies also proved the effectiveness in boosting the surface area of mesoporous carbons [28]. Accordingly, Takashi et al. also reported a record-high microporosity along with sharp pore size distribution of resulting carbons prepared from zeolite Y under nitrogen at 700 °C [29].

In this study, the Fe_3_O(BDC)_3_ was synthesized from an iron source and carboxylate via the solvothermal method, then a facile pyrolysis allowed to directly transform the Fe_3_O(BDC)_3_ into MPCs. The effect of calcination temperatures on CAP adsorption capacity was investigated to choose the best appropriate material. The materials were structurally analyzed and utilized to employ the kinetic, isotherm, thermodynamic, and recyclability studies.

## 2. Experiments

### 2.1. Chemicals and Instruments

All chemicals including chloramphenicol, 1,4–dicarboxylic acid (H_2_BDC), iron chloride FeCl_3_·6H_2_O, and potassium chloride KCl were commercially purchased from Merck. Firstly, the D8 Advance Bruker powder diffractometer was used to record the X–ray powder diffraction (XRD, Bruker, Billerica, MA, USA) profiles using Cu–Kα beams as excitation sources. The S4800 instrument (Hitachi, Krefeld, Germany) was implemented to capture the scanning electron microscope (SEM) images with the magnification of 7000 using an accelerating voltage source (15 kV). The JEOL JEM 1400 instrument (JEOL, Peabody, MA, USA) was used to study the transmission electron microscopy (TEM). The FT–IR spectra were recorded on the Nicolet 6700 spectrophotometer (Thermo Fischer Scientific, Waltham, MA, USA). The N_2_ adsorption/desorption isotherm and pore size distribution data were recorded on the Micromeritics 2020 volumetric adsorption analyzer system. The mapping element profiles were recorded on the JEOL JSM-7600F (JEOL, Tokyo, Japan). The X-ray photoelectron spectroscopy (XPS) was performed on the ESCALab MKII spectrometer (Thermo Fisher Scientific, Waltham, MA, USA) using Mg Kα radiation. The UV–Vis spectrophotometer (Shimadzu, Kyoto, Japan) was used to determine the CAP concentration at 278 nm.

### 2.2. Synthesis of Fe_3_O(BDC)_3_ and MPCs

The Fe_3_O(BDC)_3_ was solvo-thermally prepared with a slight modification [30]. In a typical experiment, 0.33 g of FeCl_3_·6H_2_O and 0.2 g of 1,4-benzenedicarboxylic acid H_2_BDC were completely dissolved in a mixture of DMF (60 mL) and ethanol (60 mL). The mixture was then transferred into two Teflon-lined autoclaves (100 mL), and heated up at 85 °C for 48 h. The solid was refluxed in DMF for 4h to remove the residual H_2_BDC. The precursor was exchanged with C_2_H_5_OH (3 × 10 mL), dried, and stored in a desiccator. 

The MPCs were fabricated using a direct pyrolysis under N_2_ nitrogen and denoted as MPCs-*x*, which *x* represents pyrolysis temperature (i.e. MPC700). The Fe_3_O(BDC)_3_ 0.8 g was carefully embarked on a heat-resistant vessel and pyrolyzed at *x* °C (*x* = 600, 700, 800, 900) for 4 h under N_2_ (100 cm^3^/min). The as-synthesized black solid was stored in a desiccator.

### 2.3. Experimental Batches

The adsorption experiments were performed in 250 mL flasks at room temperature. For the adsorption kinetics, the adsorbents (0.1 g/L) were mixed with 50 mL of CAP solutions (10 mg/L). The flasks were sealed and placed in the shaking tables (200 rpm). After the regular time intervals (30, 60, 120, 240 and 360 min), sample concentrations were analyzed using UV-Vis spectroscopy (Shimadzu, Kyoto, Japan). With respect to adsorption isotherms, the similar procedure was employed at various CAP concentrations (10, 20, 30, and 40 mg/L) at the equilibrium of 240 min. The percentages of removal *H* (%) and adsorption capacity *q* (mg/g) were calculated using the following equations (Equations (1)–(3)):

(1) $$H\left(\%\right)=\frac{C_{o}-C_{e}}{C_{o}}\cdot 100$$

(2) $$q_{t}=\frac{C_{o}-C_{t}}{m}\cdot V$$

(3) $$q_{e}=\frac{C_{o}-C_{e}}{m}\cdot V$$

where, *C_o_*, *C_t_*, and *C_e_* are initial, time *t* (min) and equilibrium concentrations (mg/L), respectively; *m* (g) and *V* (mL) are the amount of adsorbent and volume of solution, respectively.

### 2.4. Determination of pH_pzc_ (pH Point of Zero Charges)

The pH_pzc_ values were determined according to a previous report [31]. In a typical experiment, 5.0 mg of materials were poured into six flasks containing 25 mL of KCl 0.1 mol/L at the different pH values (pH_1_ = 2, 4, 6, 8, 10, 12) adjusted using the HCl and NaOH solutions. The solutions were stirred for 10 min and maintained stable during 24 h. The solids were then separated from the mixture, and their final pH_2_ were measured by a pH meter. The curve was plotted via pH_2_ versus pH_1_ and the pH_pzc_ was visualized at pH_1_ = pH_2_.

### 2.5. Error Analysis

In the nonlinear kinetic and isotherm studies, error functions could be applied for the optimization process to compare the fitness between experimental and calculated data. Herein, three common error analysis functions were utilized to assess the nonlinear models including coefficient of determination (R^2^), mean relative error (MRE), sum square error (SSE) in Equations (4)–(6). The kinetic and isotherm parameters were identified by minimizing the error functions over using the Origin^®^ 9.0 software (Originlab, MA, USA) [32]. Note that *Q_i,cal_* and *Q_i,exp_* were the theoretical and experimental values, respectively.

(4) $$R^{2}=\frac{\sum_{i=1}^{n}{\left(Q_{i,\exp}-\bar{Q_{i,\exp}}\right)}^{2}-\sum_{i=1}^{n}{\left(Q_{i,\exp}-\bar{Q_{i,cal}}\right)}^{2}}{\sum_{i=1}^{n}{\left(Q_{i,\exp}-\bar{Q_{i,\exp}}\right)}^{2}}$$

(5) $$MRE(\%)=\frac{100}{n}\sum_{i=1}^{n}\left|\frac{Q_{i,cal}-Q_{i,\exp}}{Q_{i,\exp}}\right|$$

(6) $$SSE=\sum_{i=1}^{n}{\left(Q_{i,cal}-Q_{i,\exp}\right)}^{2}$$

## 3. Results and Discussion

### 3.1. Characterization of Fe_3_O(BDC)_3_ and MPC700

**Figure 2:**
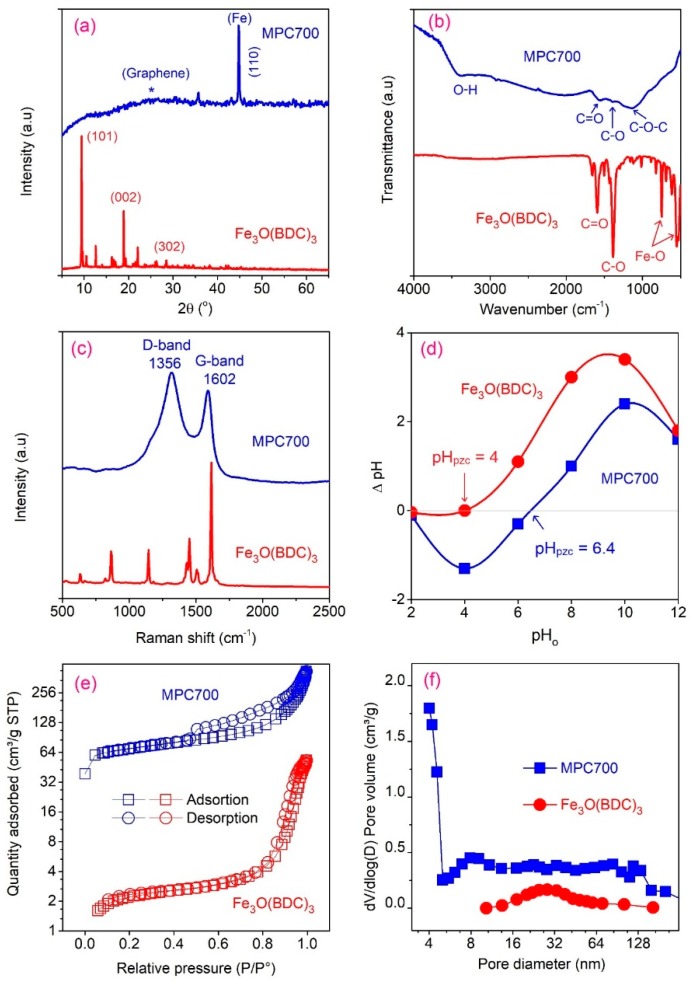
The XRD (**a**), FTIR (**b**), Raman (**c**), pH_pzc_ (**d**), nitrogen adsorption/desorption (**e**), and pore size distribution (**f**) of Fe_3_O(BDC)_3_ and MPC700.

The MPC700, which was formed by the pyrolysis of Fe_3_O(BDC)_3_ at 700 °C, was chosen as a representative to analyze the characterization along with its precursor Fe_3_O(BDC)_3_. Initially, the Fe_3_O(BDC)_3_ and MPC700 were characterized by XRD and FT-IR spectra as shown in Figure 2a,b. For the XRD profiles, the crystalline structure of Fe_3_O(BDC)_3_ showed the typical peaks at around 9.6° (101), 18.6° (002) and 28.1° (302), which matched well with previous papers [30,33]. Meanwhile, the diffraction profile for MPC700 indicated the presence of zero-valent iron (JCPDS No. 65–4899) at around 45° (110) [6]. Moreover, the diffraction region at 20–30° indicates the π-stacking of the nano-graphitic platelets of the graphite sheets. The formation of zero-valent iron (ZVI) encapsulated on MPC700 can be explained through the physical pyrolysis and in situ chemical reduction (ISCR) [34]. In fact, the calcination of Fe_3_O(BDC)_3_ at 700 °C allows to remove the volatile components e.g. H_2_O, and gradually generate the hierarchical carbon multilayers by deconstructing the aromatic rings [26]. The chemical reduction followed is expected to crack the Fe-O coordination bonds, and create the zero-valent Fe by ISCR process, proceeding in assembling the sort of carbon-encapsulated iron composite [35]. 

The surface chemistry of adsorbents involving Fe_3_O(BDC)_3_ and MPC700 could be diagnosed using the FT-IR spectra profiles as illustrated in Figure 2b. In general, the Fe_3_O(BDC)_3_ showed its typical functional groups including conjugated ketones C=O (1666 cm^−1^), and C–O (1380 cm^−1^) chemical bonds of carboxylate, which were consistent with footprints reported by previous publications [21,36]. Also, the existence of coordination bonds Fe–O (744 cm^−1^) and Fe_3_(μ_3_–O) (540 cm^−1^) could be confirmed clearly, revealing the formation of Fe (III) clusters with carboxylate groups of H_2_BDC ligands [21,37,38]. Meanwhile, although MPC700 repeated almost typical peaks as mentioned on Fe_3_O(BDC)_3_, the absence of important footprints of Fe (III)–O at the respectively low wavenumbers convinced that the ISCR process via reduction of Fe(III) (Fe_3_O(BDC)_3_) to zero-valent Fe (MPC700) is highly likely to be proceeded [22]. Therefore, this observation was again commensurate with the XRD profile evidence mentioned of the existence of zero-valent Fe on the MPC700.

The Raman spectra and pH of point zero charge curves can be used to identify more properties of materials and their profiles are shown in Figure 2c,d. The Raman spectra in Figure 2c reveals the bonds of aromatic C–H (870 cm^−1^), C–C (1160 cm^−1^), COO– (1450 cm^−1^), and C=C vibrations (1610 cm^−1^) on the Fe_3_O(BDC)_3_, which are familiar with the previous publications [39]. In the meantime, the graphitic nature of MPC700 structure can be exposed by the presence of typical D- (1330 cm^−1^) and G- (1600 cm^−1^) bands, suggesting the structurally hierarchical, amorphous, and disordered phase of materials (I_D_/I_G_ = 1.61). Meanwhile, according to Figure 2d, the pH_pzc_ values were found to be 4.0 and 6.4 for Fe_3_O(BDC)_3_ and MPC700. Note that the surface of materials tends to be more negative if the pH solution surpassed the pH of point zero charge and more positive at pH < pH_pzc_. These values are vital to explain the adsorption mechanisms [31]. 

**Figure 3:**
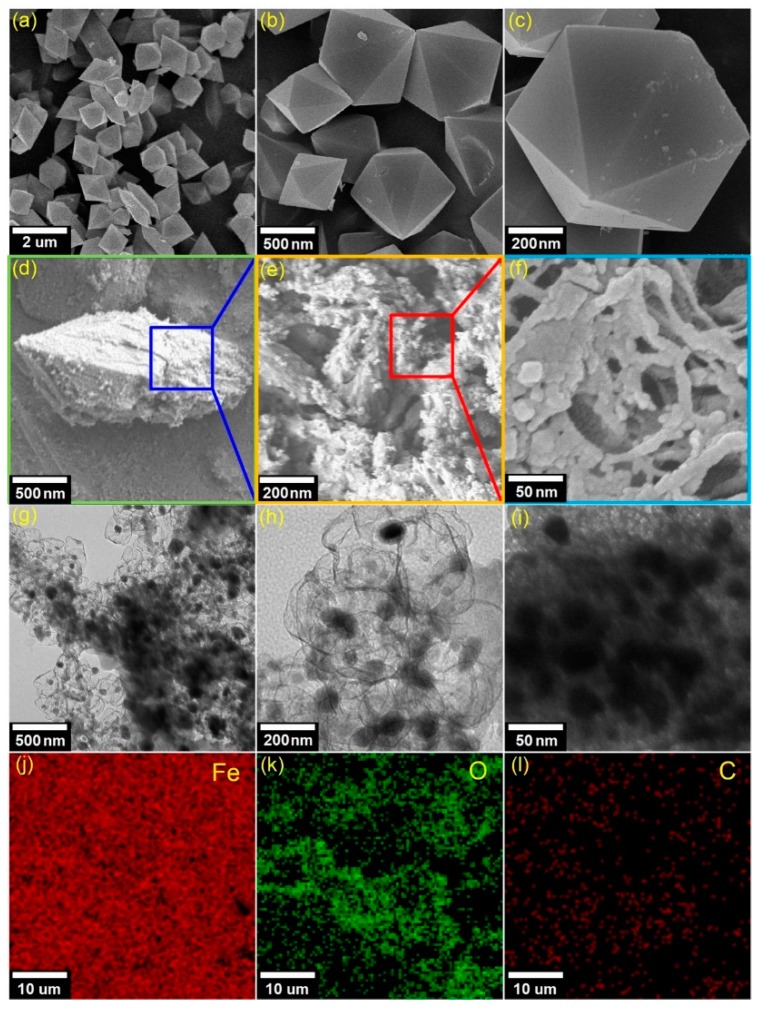
SEM images of Fe_3_O(BDC)_3_ (**a**–**c**) and MPC700 (**d**–**f**); TEM (**g**–**i**), and EDS mapping (**j**–**l**) images of MPC700.

Figure 2e,f plotted the profiles of N_2_ adsorption/desorption isotherm and pore distribution curves of Fe_3_O(BDC)_3_ and MPC700. In Figure 2e, the shape of isotherm plot for MPC700 is relatively corresponding to Type IV (IUPAC) with the presence of a hysteresis loop at high P/P_o_ ratio, indicating that MPC700 possessed the dominance of mesoporous structure. Meanwhile, the figure for Fe_3_O(BDC)_3_ seems to show the similarity to Type II (IUPAC), demonstrating the non-porous or macroporous (>50 nm in diameter) structure. Figure 3f showing the pore distribution curves of Fe_3_O(BDC)_3_ and MPC700 also supported these observations. Herein, the Brunauer-Emmett-Teller (BET) surface area values made a great difference, which was found to be 224.7 m^2^/g (MPC700) compared with 7.6 m^2^/g (Fe_3_O(BDC)_3_). A very low value of specific surface area of Fe_3_O(BDC)_3_ may be attributable to anhydrous form of Fe_3_O(BDC)_3_ exhibits closed pores with almost no accessible porosity to N_2_ at 77 K [21]. Finally, Table 2 summarized the important properties of MPC700 and Fe_3_O(BDC)_3_ including BET surface area, pore diameter, and saturation magnetization (Ms) values. With dominant favorability of surface area, porous structure, pore size, and functional groups, MPC700 was expected to show more efficient adsorption and favorable separation than Fe_3_O(BDC)_3_.

The morphological properties of the Fe_3_O(BDC)_3_ and MPC700 can be analyzed by the SEM, and TEM as shown in Figure 3. Overall, there was a morphologically noticeable difference between Fe_3_O(BDC)_3_ precursor and MPC700. In detail, the Fe_3_O(BDC)_3_ crystals in Figure 3a–c are likely to adhere to a perfect hexagonal structure at scale 500 nm, while the MPC700 obtained the relatively defective, amorphous, and heterogeneous structure as seen from Figure 3d–f. To better understand of structure of MPC700, TEM images in Figure 3g–i reveal a great dispersion of dark sites, which may be attributable to the aggregation of magnetic Fe particles and encapsulated by opaque regions (mesoporous carbon). In addition, the EDS elemental mapping in Figure 3j–l implies that structure of MPC700 was constructed by iron, carbon and oxygen elements [40].

**Figure 4:**
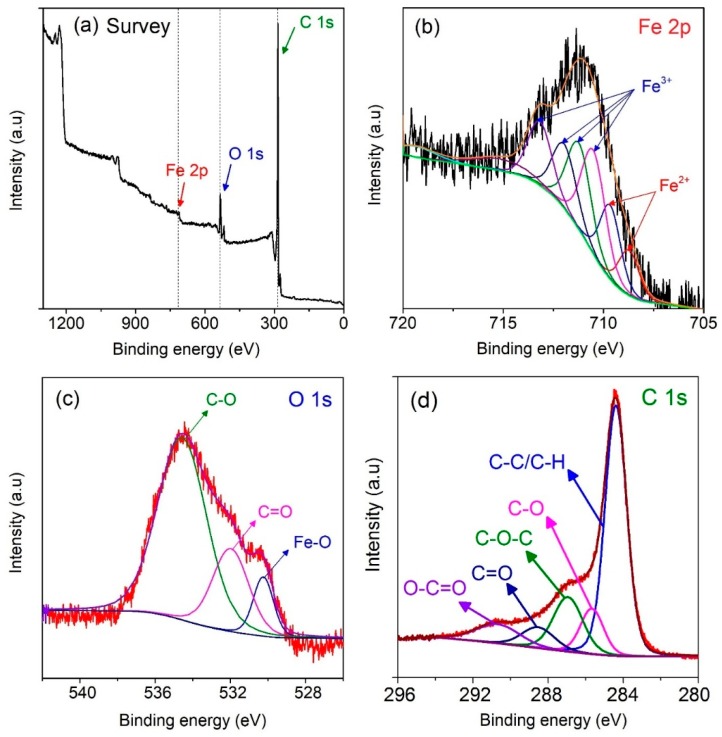
The XPS spectrum of MPC700 material: survey (**a**); Fe 2p (**b**); O 1s (**c**), and C 1s (**d**).

To gain the insight into the existence of chemical bonds in MPC700 nanocomposite, the XPS technique was further studied, and depicted in Figure 4. As observed from Figure 4a, the preliminary survey results indicate that MPC700 was solely constituted of three elements: iron (Fe 2p), carbon (C 1s) and oxygen (O 1s). For details, the Figure 4b shows the broad photoelectronic peak of Fe 2p^3/2^ sub level at around 710.8 eV. This level can be converted into various signals of Fe^3+^ species at 710.6, 711.3, 712.1, 713.3 eV, and Fe^2+^ species at 709.7, 708.8 eV, suggesting that the composite may contain both Fe^2+^ and Fe^3+^ species (FeO, Fe_3_O_4_, Fe_2_O_3_, FeOOH) without detecting any trace of ZVI [41,42,43]. However, according to the XRD and FT-IR spectra, there was the only diagnostic peak of ZVI found in the structure of MPC700. Hence, it is possible that Fe^2+^ and Fe^3+^ species encapsulate the outside shell of ZVI particles, leading to no detection of ZVI signals in the XPS spectrum of MPC700 composite because of its sensibility at limited range of depth (<10 nm) [44]. This observation also supported again the fact of the partial reduction of Fe-O bonds by carbon during the pyrolysis. Moreover, The O 1s XPS spectrum in Figure 3c exhibits three peaks at binding energies 534.6, 533.0, 530.0 eV, corresponding to chemisorbed O, C-O/C=O, and iron oxides Fe-O, while the C 1s XPS spectrum in Figure 3d indicates the presence of chemical bonds consisting of O-C=O (290.6 eV), C=O (288.5 eV), C-O-C (286.9 eV), C-O (285.7 eV), C-C/C-H (284.4 eV) [45]. These chemical bonds along with respective energies from the fitting of C 1s is very much in line with recent work [46].

Finally, Table 3 shows the quantity of functional groups on MPC700 including carboxylic (1.1 mmol/g), lactonic (0.5 mmol/g), phenolic (0.7 mol/g), and total basic (0.85 mmol/g) groups via Boehm titration. These functional groups may play a crucial role in the adsorption of CAP in aqueous solutions.

### 3.2. Adsorption Experiments

#### 3.2.1. Effect of Pyrolysis Temperature 

**Figure 5:**
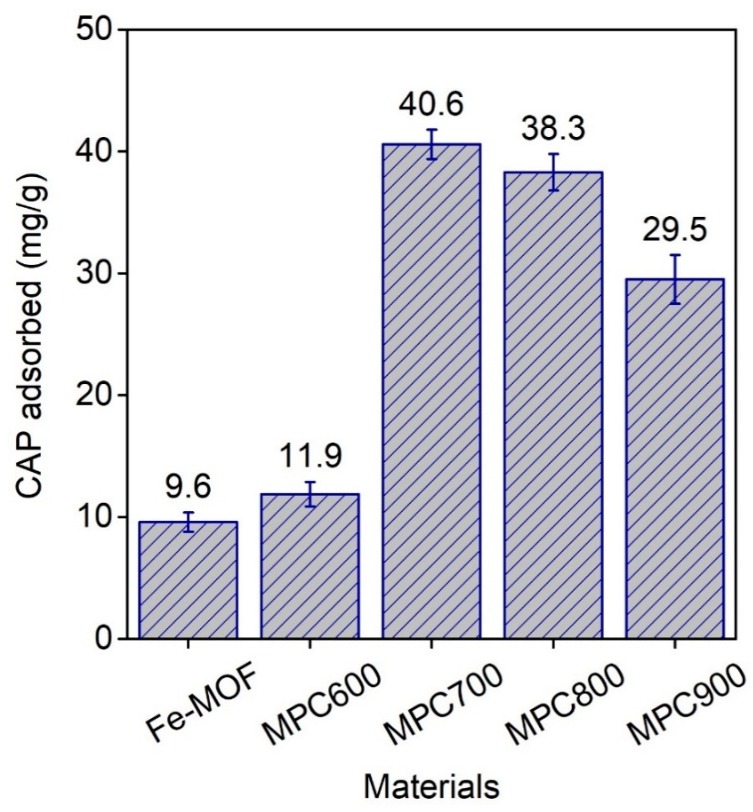
Comparative adsorption capacities of CAP antibiotic onto materials Fe_3_O(BDC)_3_ and MPCs-*x*, where *x* presents pyrolysis temperature at 600, 700, 800, and 900 °C.

The pyrolysis temperature makes a vital contribution to the formation of the structure and novel properties of materials MPCs. Herein, we investigated the effect of calcination temperature from 600 °C to 900 °C on adsorption capacity, aimed at discovering the optimal temperature for CAP adsorption. The experiments were carried out under standard conditions, but the pH values of initial CAP concentrations were unadjusted. Figure 5 illustrated the CAP adsorbed on the Fe_3_O(BDC)_3_ and MPCs-*x*. It is evident that CAP adsorption capacity of MPCs-*x* was noticeably higher than that of Fe_3_O(BDC)_3_, and MPC700 obtained the best adsorption capacity, at 40.6 mg/g, compared with 9.6 mg/g of Fe_3_O(BDC)_3_. Therefore, the arrangement of relative adsorption capacity of materials in this study complied with the order: Fe_3_O(BDC)_3_ < MPC600 < MPC900 < MPC800 < MPC700. These results proposed that MPC700 could be a delegate of the MPC family for comparing their remediation efficiency of the selected pharmaceutical CAP with pristine Fe_3_O(BDC)_3_ in the following experiments.

#### 3.2.2. Effect of Contact Time 

**Figure 6:**
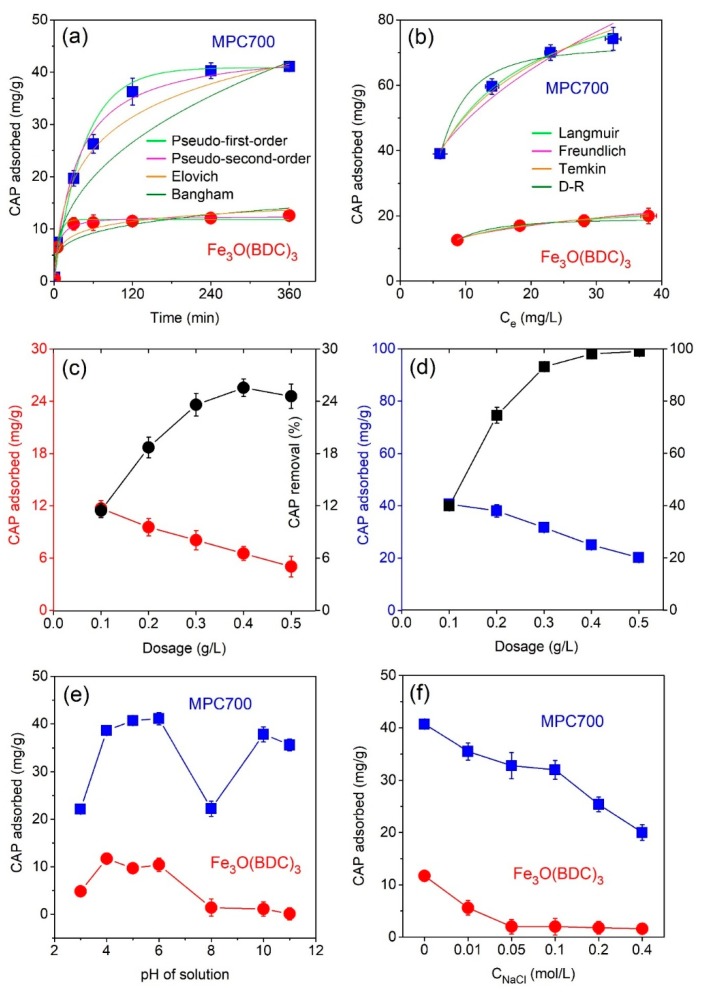
Effect of contact time (**a**); CAP concentration (**b**); adsorbent dosage (**c**,**d**); pH solution (**d**); and ionic strength (**e**) on the CAP adsorption of Fe_3_O(BDC)_3_ and MPC700.

The performance of the adsorbents including Fe_3_O(BDC)_3_ and MPC700 was surveyed within 360 min. As observed from Figure 6a, the adsorptive removal of MPC700 reached the equilibrium nature for 240 min while that tendency for Fe_3_O(BDC)_3_ was rapidly established, at only 60 min. At the equilibrium time, the CAP adsorbed quantities for Fe_3_O(BDC)_3_ and MPC700 were found to be different significantly, at 12.6 mg/g and 41.1 mg/g, respectively. 

Since the kinetic factors are likely to control the adsorption process, investigated data can be built up various models being able to characterize the adsorption behaviors and mechanisms in heterogeneous surface phases. Kinetics models including pseudo first-order, pseudo second-order, Elovich and intra-particle diffusion were simulated based on the experimental and calculated data. The compatibility of modellings could be assessed through correlation coefficient R^2^, hence the best fitting equation was chosen to justify the mechanisms of the adsorption [47]. 

Table 4 listed the equations, parameters, and error analysis methods obtained for nonlinear adsorption models of Fe_3_O(BDC)_3_ and MPC700. Based on the magnitude of adjusted R^2^ obtained, the pseudo second-order equation was found to be the most appropriate model to describe the adsorption mechanisms since their R^2^ values reached the top points, at 0.9928 and 0.9976 for Fe_3_O(BDC)_3_ and MPC700, respectively. Furthremore, the other error analysis values such as MRE (3.02–6.74%) and SSE (1.01–6.77) were very low, suggesting that the highest fitness amongs proposed kinetic models. Accordingly, the pseudo second-order equation obtained the excellent compatibility between actual and calculated data and could be used to interpret the adsorptive mechanism via chemisorption routes with rate-controlling steps [48]. 

Note that this chemisorption process is established based on electrostatic attraction between adsorbent sites and adsorbate molecules, mainly occuring on the surface functional groups. Indeed, kinetic adsorption capacity on the MPC700 (44.93 mg/g) calculated from pseudo second-order equation was so far higher than that on the Fe_3_O(BDC)_3_ (12.59 mg/g). To elucidate the effects of the heterogeneous diffusion process in liquid/gas phase on the absorbability of materials, the Elovich and Bangham equations can be explored. Due to the relatively high R^2^ value as shown in Table 4, adsorption process of CAP antibiotic molecules onto Fe_3_O(BDC)_3_ and MPC700 is well described as an intra-particle heterogeneous diffusion of CAP molecules on the mesopores, but not the only rate-controlling step [49]. Meanwhile, diffusion mechanism proposed by Elovich model reveals that adsorption rates were more rapid than desorption rates for both adsorbent objects.

#### 3.2.3. Effect of CAP Concentration 

The adsorption isotherms and respective parameters of nonlinear models involving Langmuir, Freundlich, Temkin, and Dubinin-Radushkevich (D-R) were presented in Figure 6b and summarized in Table 5. Obviously, adsorption capacities of CAP antibiotic on the Fe_3_O(BDC)_3_ and MPC700 increased with rising the initial CAP concentrations from 10 mg/L to 40 mg/L. The parameters obtained from isotherms showed the excellent adjusted coefficients of correlation R^2^ for all models (R^2^ > 0.92); and relatively low MRE values (≤11.62). Based on the (R_adj_)^2^ values, the fitness of models can be ordered as follows: Langmuir > Temkin > D-R > Freundlich for both materials. As a result, the Langmuir equation was found to be the most adequate model with R^2^ value from 0.9925 to 0.9974. These results indicated that the adsorption process adhered to monolayer mechanism, and the maximum adsorption capacity Q_m_ (mg/g) can be computed from the Langmuir equation at 96.3 mg/g and 24.1 mg/g for MPC700 and Fe_3_O(BDC)_3_, respectively. 

Moreover, the magnitude of exponent values (1/n) calculated by Freundlich equation was ranged between 0.3156 and 0.4033, while R_L_ constants calculated by Langmuir equation were found to be from 0.1639 to 0.1813, indicating that the adsorption of CAP on the Fe_3_O(BDC)_3_ and MPC700 was a favorable process. Herein, the characteristic of surface area and maximum adsorption capacities of CAP on the various magnetic materials were compared and presented in Table 6. The result of BET surface area and maximum adsorption capacity of MPC700 obtained from this study was so far higher than those reported from several previous publications. It is therefore expected that MPC700 can be a promising magnetic material for the removal of CAP antibiotic from wastewater.

#### 3.2.4. Effect of Adsorbent Dosage

Practically, the dosage of any adsorbent used directly relates to cost-effectiveness and performance of antibiotic treatment process, thus finding out the optimal dosage play a key role in overall adsorption progress. Accordingly, the amount of Fe_3_O(BDC)_3_ and MPC700 was investigated in a range of 0.1 and 0.5 g/L, while other parameters were set up at the standard conditions, e.g. CAP concentration (10 mg/L) and pH = 4 at room temperature.

As observed in Figure 6c,d, in general, when adding more adsorbent into CAP solutions, adsorption capacities of CAP antibiotic on the Fe_3_O(BDC)_3_ and MPC700 gradually decreased, coincided with an increase in removal percentage of CAP. Apparently, the higher MPC700 dosage was, the better removed CAP was. For example, nearly 100% CAP was eliminated from water if MPC700 (0.5 mg/L) was poured as shown in Figure 6c. Meanwhile, the results from Figure 6d indicate the optimal Fe_3_O(BDC)_3_ dosage of 0.4 g/L, just giving the highest removal percentage of 25.6%. 

#### 3.2.5. Effect of pH and Ionic Strength

The formation of surface charge on Fe_3_O(BDC)_3_ and MPC700, as well as ionization of CAP molecules, is dependent on the pH of the solution. Overall, the change of pH values strongly affects the adsorption capacity and removal efficiency of adsorption processes. Thus, these experiments were herein conducted in a range of 3 and 11 in pH to afford the insight into adsorption mechanisms in water. The acidity and basicity of CAP solution can be adjusted by the NaOH and HCl solutions. The Figure 6e shows the effect of pH on the CAP antibiotic absorbed on the Fe_3_O(BDC)_3_ and MPC700, which the highest adsorption capacities were found to be 11.7 and 40.8 mg/g at the optimal pH values 4.0 and 6.0, respectively. 

In detail, the adsorption capacity of CAP onto MPC700 was very low at a strongly acidic solution (pH ≤ 3), then rapidly reached the peaks in weakly acidic solution (pH 4–6). In fact, the adsorption became unfavorable at very low pH (i.e. pH = 3) due to the electrostatic repulsion effect between positively charged CAP molecules and adsorbents surface [4]. Next, this trend fell considerably when pH solution became more neutral. For example, the adsorption uptake at pH 8 was as equal as that parameter at pH 3 determined at only 22.2 mg/g, which was highly commensurate with several recent reports using persulfate activated by Fe^2+^/Fe and plasma modified steel shavings for the removal of CAP antibiotic [4,5]. Finally, the adsorption capacity of CAP onto MPC700 again witnessed the noticeable recovery at pH 10 and 11. This abnormality could be attributed by an “accumulation of solutes” mechanism, which the dihydroxylation of CAP under higher pH may create a positively charged CAP, resulting in an electrostatic attraction with a negatively charged surface of MPC700 [4]. In comparison with the adsorption of CAP onto MPC700, that process onto Fe_3_O(BDC)_3_ was more stable regarding pH changes. As seen from Figure 6e, the adsorption of CAP onto Fe_3_O(BDC)_3_ is favorable in acidic solution, while variation by augmenting the pH values caused the insignificant fluctuation in the adsorption capacity of CAP. 

The inorganic salts are the important components that often exist in water, thus affect the adsorption mechanism through enhancing/restraining the solubility of CAP and the electrostatic interactions between adsorbent and adsorbate. Herein, the sodium chloride NaCl was simulated as the inorganic salt to measure the effect of salinity on CAP adsorption on the Fe_3_O(BDC)_3_ and MPC700. The NaCl concentrations were identified from 0 to 0.4 g/L by adding the amount of dehydrated NaCl into CAP solutions. As observed from Figure 6f, adsorption capacity measured for both adsorbents generally decrease with increasing in NaCl concentration. At the NaCl concentration higher 0.05 mol/L, there was a negligible change in adsorption uptake onto Fe_3_O(BDC)_3_, while the figure for that onto MPC700 considerably decreased. At NaCl 0.4 g/L, only 20.3 mg/g and 1.6 mg/g of CAP absorbed onto MPC700 and Fe_3_O(BDC)_3_, respectively. The decrease of adsorption capacity of CAP onto materials at more concentrated solutions of NaCl can be attributable to “salting-out” effect as NaCl is solvated by H_2_O then forming Na^+^ and Cl^-^ ionic species. These ions control the CAP solubility in water, therefore adding more amount of NaCl decreased the solubility of CAP in water and increased the competition between CAP^+^ and Na^+^ on the surface of the material. Finally, the adsorption capacity of CAP decreased in the presence of NaCl salt.

### 3.3. Thermodynamic and Recyclability Studies 

The thermodynamic study shows the effect of temperature on the adsorption of CAP, and give the prediction about whether the adsorption is a spontaneous process or not. Their standard parameters could be represented as follows (Equation (7)):

(7) $$\Delta G=-RT\ln K_{C}$$

where, *K*_C_ and *T* (K) are the adsorption equilibrium constant and temperature, respectively. *K*_C_ can be calculated through the ratio of equilibrium concentration of adsorbent between liquid and solid phase and determined as follows (Equation (8)):

(8) $$\ln K_{C}=\frac{C_{A}}{C_{e}}$$

where, *C_A_* (mg/g) and *C_e_* (mg/L) are the equilibrium CAP concentrations in solid phase and solution phase, respectively [50]. Standard enthalpy (*ΔH*) and entropy (*ΔS*) can be calculated by van’t Hoff isotherm equation as follows (Equation (9)):

(9) $$\ln K_{C}=\left(\frac{-\Delta H}{R}\right)\cdot \frac{1}{T}+\frac{\Delta S}{R}$$

**Figure 7:**
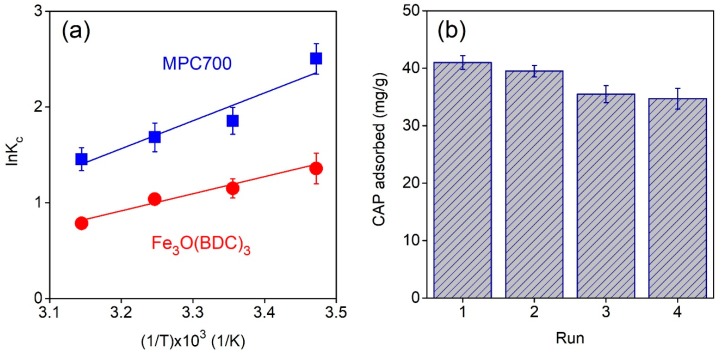
Thermodynamic (**a**) and recyclability (**b**) studies.

Figure 7a plots the impact of temperature (288–318 K) on CAP adsorption onto MPC700 and Fe_3_O(BDC)_3_. In addition, thermodynamic constants involving enthalpy (*∆H*), entropy (*∆S*) and Gibbs free energy (*∆G*) were shown in Table 7. The negative *∆H* values indicate the adsorption of CAP over MPC700 and Fe_3_O(BDC)_3_ was an exothermic process, which totally agreed with a recent work [51]. Meanwhile, negative values of *∆S* show a decline in disorder occurring in heterogeneous phase because of migration between solvent and CAP molecules during sorption [45]. Finally, the negative values of Gibbs free energy indicate that the adsorption of CAP onto MPC700 and Fe_3_O(BDC)_3_ was a spontaneous process.

One of the most important properties of adsorbent relating to cost-effectiveness and practical applicability is their stability and recyclability [52]. In this study, adsorbent MPC700 can be regenerated by using a green eluent mixture of methanol/acetic acid (9:1) [53]. The regeneration procedure could be described as follows: The CAP@MPC700 was rapidly separated using a magnetic field, washing with a mixture of eluents (3 × 10 mL), drying and reactivating under 110 °C for 4 h. As shown in Figure 7b, after for cycles, there was a slight decline in the CAP adsorption capacity from 41.0 mg/g to 34.7 mg/g, suggesting good stability and regeneration performance of MPC.

### 3.4. Proposed Mechanism 

As mentioned, the maximum adsorption capacity values of CAP by MPC700 was so far higher than that by Fe_3_O(BDC)_3_. These results reflected the importance of surface functional groups towards adsorption of CAP. According to Boehm titration in Table 3, functional groups including carboxylic, phenolic, and lactonic groups on the surface of MPC700 were found, which provide an abundance of oxygen atoms or hydroxyl groups. Meanwhile, each CAP molecule contains two alcoholic groups that can readily create a type of H-bond with these oxygen atoms or hydroxyl groups. As a result, there was the existence of H-bond in adsorption mechanism.

**Figure 8:**
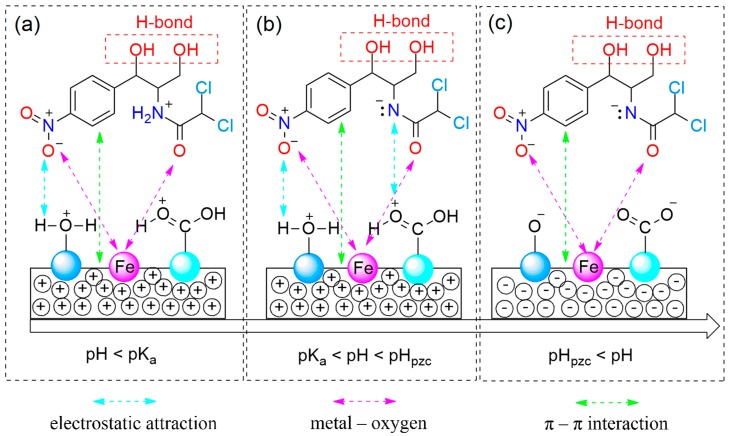
Proposed mechanism: (**a**) pH < pK_a_; (**b**) pK_a_ < pH < pH_pzc_; and (**c**) pH_pzc_ < pH.

However, the sorption of CAP onto materials can occur at any pH values possibly due to the existence of other factors such as electrostatic attraction, π–π interaction, and metal–bridging interaction (Figure 8). While the electrostatic attraction is influenced by the pH values, the kind of latter interactions may be independent on acidity or basicity, making decision on whether the adsorption process in aqueous solution is favorable or not. 

Obviously, the π-electronic rich system on mesoporous carbon of MPC700 can create a special interaction with π-electrons of aromatic rings on CAP molecules, called “π–π interaction”, while the bridge between zero-valent iron and oxygen-containing functional groups results in the type of metal-oxygen interaction, as observed in Figure 8a–c. Otherwise, the electrostatic attraction can be divided into three circumstance. 

Firstly, at pH < (pK_a_)_CAP_ in Figure 8a, the surface functional groups on MPC700 and CAP molecules are all protonated in high acidic solution. Therefore, the formation of electrostatic attraction between negative and positive sites becomes more difficult. The adsorption in this case is not dominant. 

Secondly, at (pK_a_)_CAP_ < pH < pH_pzc_ in Figure 8b, while the surface of MPC70 is positively charged (pH < pH_pzc_ = 6.4), the CAP molecules is mainly deprotonated (pH > (pK_a_)_CAP_ = 5.5), resulting in the prevalent presence of negative and positive sites. Thus, the electrostatic attraction is permitted to run, and adsorption of CAP is a favorable process. In fact, the results in Figure 6e also indicated the best condition for the adsorption of CAP at 6.0, which was highly consistent with given statement. 

Thirdly, at pH_pzc_ < pH in Figure 8c, both the surface of MPC700 and CAP molecules are all deprotonated in high basic solution (pH > 6.4), and hence, electrostatic attraction is exterminated, leading to a detrimental adsorption process.

## 4. Conclusions 

In this study, the Fe_3_O(BDC)_3_ and MPC700 materials were successfully synthesized via the solvothermal method. The materials were characterized by XRD, FT-IR, SEM, TEM, BET and VSM techniques, indicating that Fe_3_O(BDC)_3_ revealed the high crystalline structure while MPC700 existed the mesoporous carbon coating the zero-valent Fe. The effect of pyrolysis temperature was investigated, confirming that MPC700 inherited the highest adsorption capacity of CAP amongst MPCs-*x* materials. To compare the absorbability of MPC700 with Fe_3_O(BDC)_3_, adsorption kinetics and isotherms were also studied. The results indicated that the pseudo second-order and Langmuir equations were the most suitable models to describe the monolayer chemisorption of CAP onto materials based on the correlation of determination R^2^. With high maximum adsorption capacity of 96.3 mg/g, the MPC700 is recommended to be an efficient adsorbent for removing the chloramphenicol in water.

## Figures and Tables

**Table 1:** Several properties of chloramphenicol (CAS No. 56-75-7).

Chemical Structure	Log Kow [5]	pK_a_ in Water (25 °C) [5]	Wavelength (nm)	Number of H-Bond
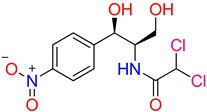	1.14	5.5	278	9

**Table 2:** Characteristics of Fe_3_O(BDC)_3_ and mesoporous carbon (MPC)700.

Materials	*S*_BET_ (m^2^/g)	Total Pore Volume (cm^3^/g)	Pore Diameter (Å)	pH_pzc_	Ms (emu/g)
Fe_3_O(BDC)_3_	7.6	0.01	27.4	4.0	0
MPC700	224.7	0.14	12.2	6.4	6.3

**Table 3:** Surface groups obtained from Boehm titration of Fe_3_O(BDC)_3_ and MPC700.

Materials	Acidic Groups (mmol/g)				Total Basic Groups (mmol/g)
	Carboxylic	Lactonic	Phenolic	Total	
Fe_3_O(BDC)_3_	-	-	-	-	-
MPC700	1.1	0.5	0.7	2.3	0.85

**Table 4:** Kinetic constants for the adsorption of CAP by Fe_3_O(BDC)_3_ and MPC700.

Kinetic Models	Equation	Parameters	Fe_3_O(BDC)_3_	MPC700
Pseudo first-order	$Q_{t}=Q_{1}\cdot \left(1-\exp(-k_{1}t)\right)$	k_1_ (min^−1^/(mg/L)^1/n^)	0.1150	0.0228
		Q_1_ (mg/g)	11.86	40.98
		MRE (%)	3.28	10.11
		SSE	0.83	8.01
		(R_adj_)^2^	0.9905	0.9954
Pseudo second-order	$Q_{t}=\frac{t}{\frac{1}{k_{2}Q_{2}^{2}}+\frac{t}{Q_{2}}}$ $H=k_{2}\cdot Q_{2}^{2}$	k_2_ (g/(mg.min))	0.0106	0.000681
		Q_2_ (mg/g)	12.59	44.93
		H	1.6753	1.3759
		MRE (%)	3.02	6.74
		SSE	1.01	6.77
		(R_adj_)^2^	0.9928	0.9976
Elovich	$Q_{t}=\frac{1}{\beta }\ln(1+\alpha \beta t)$	α (mg/(g·min))	3.8433	2.5749
		β (g/mg)	0.4843	0.1119
		MRE (%)	11.04	11.72
		SSE	9.66	44.56
		(R_adj_)^2^	0.9390	0.9893
Bangham	$Q_{t}=k_{B}\cdot t^{\alpha_{B}}$	k_B_ (mL/(g/L))	3.3533	5.3828
		α_B_	0.2409	0.3484
		MRE (%)	17.35	28.65
		SSE	18.12	153.96
		(R_adj_)^2^	0.8714	0.9554

**Table 5:** Isotherm constants for the adsorption of CAP by MPC700 and Fe_3_O(BDC)_3_.

Kinetic Models	Equation	Parameters	Fe_3_O(BDC)_3_	MPC700
Langmuir	$Q_{e}=\frac{Q_{m}K_{L}C_{e}}{1+K_{L}C_{e}}$ $R_{L}=\frac{1}{1+K_{L}C_{o}}$	k_L_ (L/mg)	0.128	0.113
		Q_m_ (mg/g)	24.1	96.3
		R_L_	0.1639	0.1813
		MRE (%)	0.89	7.38
		SSE	0.16	100.43
		(R_adj_)^2^	0.9925	0.9974
Freundlich	$Q_{e}=K_{F}C_{e}{}^{1/n}$	k_F_ (mg/g)/(mg/L)^1/n^	6.62	19.36
		1/n	0.3156	0.4033
		MRE (%)	11.62	4.26
		SSE	17.38	37.09
		(R_adj_)^2^	0.9277	0.9606
Tempkin	$Q_{e}=B_{T}\ln(k_{T}C_{e})$ $B_{T}=\frac{RT}{b}$	k_T_ (L/mg)	1.3363	0.9417
		B_T_	5.2327	22.51
		MRE (%)	11.11	2.0894
		SSE	15.99	11.13
		(R_adj_)^2^	0.9695	0.99
D-R	$Q_{e}=Q_{m}\exp\left(-B\varepsilon^{2}\right)$ $\varepsilon =RT\ln\left(1+\frac{1}{C_{e}}\right)$ $E=\frac{1}{\sqrt{2B}}$	B (kJ^2^/mol^2^)	5.9580	4.43
		Q_m_ (mg/g)	19.13	72.23
		E (kJ/mol)	0.2897	0.3360
		MRE (%)	10.42	3.5971
		SSE	15.70	30.9531
		(R_adj_)^2^	0.9539	0.9722

**Table 6:** A comparison of BET surface area and adsorption capacity of adsorbents.

No.	Adsorbents	BET Surface Area (m^2^/g)	Maximum Adsorption Capacity (mg/g)	Ref.
1	MPC700	224.7	96.3	This work
2	Fe_3_O(BDC)_3_	7.6	24.1	This work
3	Sol-gel MIP	167. 3	23.0	[1]
4	Bamboo charcoal	67.8	8.1	[2]
5	Plasma modified StS (M3-plN2)	4.5617	3.167	[5]
6	Raw StS (M3)	2.7179	2.92	[5]
7	BSA/Fe_3_O_4_	-	147.83	[3]

**Table 7:** Thermodynamic parameters for the adsorption of CAP.

Parameters	Unit	Fe_3_O(BDC)_3_	MPC700
∆H	J/mol	−13,895	−25,443
∆S	J/mol·K	−36.9	−68.5
∆G_288_	J/mol	−3262.5	−5714.5
∆G_298_	J/mol	−2893.3	−030.0
∆G_308_	J/mol	−2524.2	−4344.5
∆G_318_	J/mol	−2155.0	−3660.0
$R^{2}$	-	0.9777	0.9188
